# Cytotoxicity Evaluation of Iranian Propolis and Calcium Hydroxide on Dental Pulp Fibroblasts

**DOI:** 10.5681/joddd.2014.024

**Published:** 2014-09-17

**Authors:** Maryam Zare Jahromi, Parisa Ranjbarian, Samaneh Shiravi

**Affiliations:** ^1^Assistant Professor, Department of Endodontics, Faculty of Dentistry, Islamic Azad University Khorasgan (Isfahan) Branch, Isfahan, Iran; ^2^Post-graduate Student, Department of Endodontics, Faculty of Dentistry, Islamic Azad University, Khorasgan (Isfahan) Branch, Isfahan, Iran; ^3^Dentist, Private Practice, Isfahan, Iran

**Keywords:** Cytotoxicity, calcium hydroxide, fibroblast, MTT, propolis

## Abstract

***Background and aims.*** Since intracanal medicaments can affect the cell viability in periapical tissues, the aim of this study was to evaluate the effect of calcium hydroxide and propolis on pulp fibroblasts.

***Materials and methods.*** Two healthy third molars were used as a source to obtain fibroblasts. The fibroblasts were cultured and subjected to 1 mg/mL of propolis and calcium hydroxide. This experiment was performed in six replicates and cell viability was evaluated with MTT assay. Statistical analysis was performed by t-test.

***Results.*** Comparison of cell viability with the use of 1 mg/mL of calcium hydroxide and propolis showed that cells subjected to propolis were more viable when compared to calcium hydroxide (P < 0.05).

***Conclusion.*** In this study, calcium hydroxide reduced fibroblast viability, significantly more than Iranian propolis. Other properties should be evaluated before Iranian propolis could be indicated for use as intracanal medicament.

## Introduction

Intracanal medicaments must be used at concentrations that are bactericidal while having minimal effects on cell viability in periapical tissues. The choice of this agent depends on biologic characteristics; such medicaments should be non-irritant, able to control the intensity and duration of inflammatory processes and infection, and have healing potential.^1,2^ Presently, calcium hydroxide is the most commonly used medication for root canal disinfection.^1,2^This application of calcium hydroxide is based on its ability to neutralize microbial byproducts; however, because of its high pH, calcium hydroxide can lead to chronic inflammation and cell necrosis in vivo.^3^ In addition, calcium hydroxide has shown to be ineffective in destroying bacteria in vivo.^3^ The inefficiency of calcium hydroxide is suggested to be caused by failure of the medication to reach the site of action and by the buffering capacity of hydroxyapatite.^4,5^There has been considerable discussion about finding alternate intracanal medications as an adjunct to irrigation and mechanical cleaning for the elimination of bacteria with minimum side effect and maximum biocompatibility. Recently, propolis, a flavonoid-rich product of bee wax, has shown to possess antimicrobial and anti-inflammatory properties.^7,8^ The main chemical agents present in propolis are flavonoids, phenolics and various aromatic compounds.^6^ Flavonoids are well-known plant compounds that have antioxidant, antibacterial, antifungal, antiviral and anti-inflammatory properties; they also aid the immune system by promoting phagocytic activity and stimulate cellular immunity.^7^

Other applications of propolis, other than as an intracanal medicament, in dentistry include medicament for wound healing, intermediate environment for avulsed teeth, its use as a pulp capping material, anti-cavity effect (due to antibacterial activity against *Streptococcus mutans*), treatment of denture stomatitis, tooth sensitivity control, effects and treatment of recurrent aphthous stomatitis and acute necrotizing ulcerative gingivitis.

Commercially available forms of propolis are toothpastes, mouthwashes, rod tablets, beverages, cakes, powders, gels and soap.

Awawdeh et al^8^ evaluated the efficacy of propolis and calcium hydroxide as a short-term intracanal medicament against *Enterococcus faecalis*. They concluded that propolis is very effective as intracanal medicament in rapidly eliminating *E. faecalis ex vivo*. Ahangari et al^9^ compared the antimicrobial activity of propolis and calcium hydroxide and claimed that propolis is more effective against *Enterococcus faecalis* and *Lactobacillus peptostreptococcus* than calcium hydroxide in agar culture. Victorino et al^10^ produced a propolis paste formulation for endodontic use, with the lowest concentration of crude extract of propolis which retains its biological activity. Other authors have shown that propolis can be useful as a root canal dressing due to its low toxicity and broad antibacterial spectrum.^11^ Al-Shaher et al^3^ examined the resistance of fibroblasts of the periodontal ligament (PDL) and dental pulp to propolis compared with calcium hydroxide in their in vitro study. Data revealed that exposure of PDL or pulp fibroblasts to 4 mg/mL or lower concentrations of propolis resulted in more than 75% cells viability. On the contrary, 0.4 mg/mL of calcium hydroxide was cytotoxic and less than 25% of the cells were found to be viable. In conclusion, propolis can be recommended as a suitable transport medium for avulsed teeth.^3^Further investigations may find propolis to be a possible alternative for an intracanal antimicrobial agent. The aim of this study was to investigate the effect of Iranian propolis and calcium hydroxide on pulp fibroblasts.

## Materials and Methods

### Preparation of ethanol extract of propolis (EEP)

Propolis samples were obtained from the beehives of Esfahan countryside. Propolis was minced with little hand pressure into pieces with a thickness of 2-4 mm at 37°C and then transferred to an environment with a temperature of -20°C. After 24 hours the samples were ground in an electric mill. The resultant powder was transferred to and maintained in a -20°C environment for 24 hours and then was ground again.^12^ A total of 5 grams of propolis was dissolved in 50 mL of 96% ethanol at 37ºC and sonicated for 10 minutes. The solution was filtered using a 20-µ filter; EEP was obtained at a concentration of 100 mg/mL EEP has better effects than aqueous solution due to the easier release and isolation of flavonoids (the active component of propolis).^11^

To perform this experimental study, two healthy third molars were used as a source to obtain fibroblasts; the extraction procedure was kept simple to prevent tooth damage. Immediately after extraction, the third molars were washed using gauze soaked in 70% ethanol (Zakariya Razi, Tehran, Iran), followed by a wash with sterile distilled water (Gibco,  Carlsbad, US). Holding the tooth with upper incisor forceps (Aesculap, Tuttlingen, Germany), a cut was made between the enamel and the cement using a cylindrical bur (Tizkavan, Tehtan, Iran). A fracture was made on the same line and the tooth fragments were placed in a Falcon flask containing sterile PBS 1X (Gibco, Carlsbad, US). The samples were rapidly transported to the laboratory and placed in Petri dishes in a laminar flow hood (Jal Tajhiz, Karaj, Iran). The tissues were isolated from the dental pulp using a #15 sterile endodontic file and forceps. Cellular separation was completed by digesting the divided pulp tissue with 3 mg/mL collagenase type I (Sigma, Seelze, Germany) for 60 minutes at 37°C. The cells were then separated using an insulin syringe and centrifuged for 10 minutes at 1800 rpm. The cell fraction was washed twice with sterile PBS 1X and centrifuged again for 10 minutes at 1800 rpm at room temperature.^13^ The obtained fibroblasts were cultured in DMEM #Hams F12 (Gibco,  Carlsbad, US) supplemented with 10% FBS (Sigma, Seelze, Germany), 2 ML-glutamine (Sigma, Seelze, Germany), penicillin G 100 mg/mL (Sigma, Seelze, Germany), streptomycin 100 µg/mL (Sigma, Seelze, Germany) and 1% Fungizone (Sigma, Seelze, Germany) and incubated at 37°C in humidified 95% air and 5% CO_2_for 3 weeks.^14^The medium was refreshed every 3 days until the cells reached 80% confluency.

In the third passage the cells were plated at 1×10^5^ cell/mL per well onto 96-well plates. Then the cells maintained at 37°C and 5% CO_2_ in a humidified incubator (Memmert, Frankfurt, Germany).^15^ Then 10 µL of 1 mg/mL propolis and calcium hydroxide(Merck, Frankfurter, Germany) was added to each well and incubated for 24 hours again. After exposure to the solutions, the cells were washed twice in PBS to remove dead cells.^16^To determine cell viability 100 µL of MTT [3-(4,5-dimethylthiazol-2-yl)-2,5-diphenyltetrazolium bromide] (Sigma, Seelze, Germany) solution (5 mg/mL) was added to each well of the plate and further incubated at 37°C for 4 hours. To dissolve the formazan precipitate the medium in each well was changed to 100 µL dimethyl sulfoxide (DMSO) (Sigma, Seelze, Germany) and mixed thoroughly. After 10 minutes at room temperature to ensure that all crystals were dissolved, optical densities of each plate were read with ELISA reader (Biorad, Mannheim, Germany) at a wavelength of 550 nm.^17^ Cells exposed to cell culture medium served as a control for cell viability. All the experiments were conducted in six replicates per group and statistical analysis was performed by t-test. In addition, the following grading system was used for qualitative comparison of the results: in 90% cell viability, the material was considered biocompatible; in 60-90% cell viability, it was considered mild toxic; in 30-59% moderately toxic, and in less than 30% of cell viability it was considered severely toxic.^18^

## Results

The percentage of cell viability was calculated as the relative absorbance of sample versus control wells as follows: % cell viability = (mean optical density of experimental well/mean optical density of control well) ×100. In this context, treatment of dental pulp fibroblasts with 1 mg/mL of propolis and calcium hydroxide resulted in 75.20% and 11.34% cell viability, respectively. Comparison of the cell viability showed that at concentration of 1 mg/mL, cells subjected to propolis significantly were more viable and calcium hydroxide was extremely toxic (P< 0.05)(Table 1). Thus, the cytotoxicity of calcium hydroxide and propolis in these concentrations was considered severe and mild.

**Table 1:** Statistical values ??of the optical density of 1 mg/mL of propolis and Ca(OH)<sub>2</sub>

Group	N	Mean	Std. Deviation	P value
Ca(OH)_2_	6	0.35500	0.037084	<0.001
Propolis	6	2.35400	0.117849	
Control	6	3.1300	0.199198	

## Discussion

There have been considerable discussions about finding ideal intracanal medicaments; however, to justify their application, antibacterial activity must exceed cytotoxicity. Antibacterial agents that are cytotoxic and have enough potential to eliminate bacteria may damage periapical tissues.^19^ Trevino et al^20^ in a study concluded that irrigants and intracanal medicaments greatly affect the survivability of cells within the root canal environment; therefore, evaluation of cytotoxicity of these agents beside antibacterial activity seems to be necessary. Calcium hydroxide has been introduced as an effective intracanal medicament because of its alkaline PH and antibacterial effect. However, because of high PH, calcium hydroxide is potentially toxic and tends to dissolve soft tissues.^3^ Additionally, evidence indicates that a number of microorganisms such as *E. faecalis*^21^, *Candida* species^22^ and *Actinomyces radicidentis*^23,24^are resistant to calcium hydroxide. Due to these disadvantages, the new and natural materials with minimum side effects and high antibacterial activity have attracted the attention of endodontists in endodontic therapy. One of these materials is propolis. Since calcium hydroxide is toxic and bioflavonoids in propolis are antibacterial and anti-inflammatory, and due to the low content (0.5%) of alcohol in propolis extraction, this study intended to examine the effect of propolis on the fibroblasts of dental pulp as the first step in its possible use as an alternative intracanal medication. Considering the fact that the effect of propolis varies in different geographical regions and seasons of the year, spring-extracted propolis of Iran was used. To quantify viable cells, MTT assay was used. MTT is a simple, fast and reliable method for cytotoxicity assay. The methyl-tetrazolium ring is cleared to formazan by mitochondrial dehydrogenases in viable cells; formazan is blue in color and can be measured with a spectrophotometer.^25^ The amount of formazan produced is directly proportional to the total viable cell number.^25^ In a study by Al-Shaher et al,^3^ calcium hydroxide was approximately 10 times more cytotoxic than propolis. These results are similar to our findings. Our comparison of the toxicity of propolis to calcium hydroxide showed that at concentration of 1 mg/mL of calcium hydroxide, which is the most commonly used concentration for cytotoxicity assay in similar studies,^1,26^ resulted in less cell viability than propolis. The cause of more toxicity of calcium hydroxide may be attributed to high pH and hydroxyl ions adjacent to cells, resulting in cell necrosis and apoptosis.^27^ Anti-inflammatory properties might be the causes of lower toxicity of propolis, perhaps through suppression of immune cells, activation of macrophage-derived nitric oxide and cytokine production, neutrophil activation and also synthesis of collagene.^28^ According to the results of this study and because of antimicrobial properties and ability to enhance immune response^3^and lower cytotoxicity, propolis can be considered an intracanal medicament. However, further studies should be performed in order to investigate other properties of this substance.

Limitations of this study were difficulty of finding open-apex third molars with minimal trauma and a simple way out, difficulty of ethanol extract of propolis and achieving appropriate levels of EEP, which is time-consuming and increases the cost of the study.

## Conclusion

Calcium hydroxide significantly reduced fibroblasts viability more than Iranian propolis. Therefore, perhaps after evaluating other properties, Iranian propolis might be considered an intracanal medicament.
